# Pharmacometric Analysis of Intranasal and Intravenous Nalbuphine to Optimize Pain Management in Infants

**DOI:** 10.3389/fped.2022.837492

**Published:** 2022-03-02

**Authors:** Miriam Pfiffner, Eva Berger-Olah, Priska Vonbach, Marc Pfister, Verena Gotta

**Affiliations:** ^1^Hospital Pharmacy, University Children's Hospital Zurich, Zurich, Switzerland; ^2^Emergency Unit, University Children's Hospital Zurich, Zurich, Switzerland; ^3^PEDeus, A Subsidiary of the University Children's Hospital Zurich, Zurich, Switzerland; ^4^Pediatric Pharmacology and Pharmacometrics Research Center, University Children's Hospital Basel (UKBB), Basel, Switzerland

**Keywords:** nalbuphine, opioid analgesics, pharmacokinetics, pharmacodynamics, exposure response, infants, pediatrics

## Abstract

**Objectives:**

The objective of this pharmacometric (PMX) study was to (i) characterize population pharmacokinetics (PPK) and exposure-pain response associations following intranasal (0.1 mg/kg) or intravenous (IV, 0.05 mg/kg) administration of nalbuphine, with the goal to (ii) evaluate strategies for optimized dosing and timing of painful interventions in infants 1–3 months old.

**Methods:**

PPK analysis of nalbuphine serum concentrations, prospectively collected 15, 30, and between 120 and 180 min post-dose, utilizing the software package Monolix. The final PPK model was applied to derive individual time-matched concentration predictions for each pain assessment (Neonatal Infant Pain Score, NIPS) after establishment of venous access and urinary catheterization or lumbar puncture. Drug exposure-pain response simulations were performed to evaluate potential benefits of higher doses with respect to a previously proposed target concentration of 12 mcg/L (efficacy threshold).

**Results:**

Thirty-eight of 52 study subjects receiving nalbuphine had at least one concentration measurement and were included in the pharmacometric analysis. A two-compartment model with allometric scaling was applied to describe population PK data, with intranasal bioavailability estimated to be 41% (95%CI: 26–56%). Model-based simulations showed that the proposed efficacy threshold (12 mcg/L) is expected to be exceeded with an IV dose of 0.05 mg/kg for 6 min, with 0.1 mg/kg for 30 min and with 0.2 mg/kg for 80 min. This efficacy threshold is not achieved with intranasal doses of 0.1 and 0.2 mg/kg, whereas an intranasal dose of 0.4 mg/kg is expected to exceed such threshold for 30 to 100 min.

**Conclusion:**

This PMX study confirmed that bioavailability of intranasal nalbuphine is close to 50%. Exposure-pain response simulations indicated that an intranasal dose of 0.4 mg/kg is required to provide a comparable pain control as achieved with an IV dose of 0.1–0.2 mg/kg. The optimal time window for painful procedures appears to be within the first 30 min after IV administration of 0.1 mg/kg nalbuphine, whereas such procedures should be scheduled 30 min after an intranasal dose of 0.4 mg/kg nalbuphine. Additional clinical studies are warranted to confirm these PMX based recommendations and to further optimize pain management in this vulnerable infant population.

## Introduction

Nalbuphine is an opioid analgesic agent that is used for the treatment of moderate to severe pain. Due to its unique mixed agonist and partial antagonist properties (on kappa and mu opioid receptors, respectively), it shows a lower ceiling effect on respiratory depression compared to mu receptor agonists such as morphine or fentanyl (1–3). For this reason it is frequently used in pediatric patients, including infants and neonates (4–6). Although nalbuphine has been approved more than 20 years ago, pharmacokinetic data on the drug remain limited, especially in children and optimal dosing is not well characterized in infants and young children (4–10). As the drug undergoes extensive and variable first-pass metabolism, nalbuphine is usually given parenterally (1, 4, 8). The metabolism of nalbuphine involves phase I oxidation–reduction *via* Cytochrome P450 to 25% (*via* CYP2C9 and CYP2C19) and phase II glucuronidation *via* UDP-glucuronosyltransferases to 75% (UGT2B7, UGT1A3, and UGT1A9) (11).

We have previously investigated whether intranasal administration could be a non-invasive alternative route of administration in infants 1–3 months of age for interventional pain management, as establishing venous access can be particularly time-consuming, difficult and stressful in this age group (12–16). Despite expected different pharmacokinetic profiles after intravenous (IV) and intranasal administration, we observed overall comparable tolerability and exposure coverage in terms of area under the concentration time curve during the first 2.5 h following single administration of 0.1 mg/kg intranasal vs. 0.05 mg/kg IV nalbuphine. However, a relatively high proportion of study subjects exhibited severe pain as assessed by neonatal infant pain score (NIPS), and a previously proposed target concentration of 12 mcg/L (in children >1 years under continuous infusion) (4), was achieved neither by IV nor intranasal administration in this study.

Population pharmacokinetic and exposure-pain response modeling are pharmacometric (PMX) tools that can facilitate evaluation and optimization of current dosing in young children with the goal to further improve pain control and optimize timing of interventions (17, 18). As such, key objectives of this PMX study were (i) to characterize population pharmacokinetics after single IV and intranasal administration in infants 1–3 months of age, (ii) to explore exposure-pain response associations in these pediatric patients, and (iii) to perform PMX-based simulations to evaluate and optimize dosing strategies in this vulnerable pediatric patient population.

## Trial Design, Participants and Interventions

Data used for this analysis originates from a prospective, single center, open-label pharmacokinetic and safety study performed in the interdisciplinary emergency department at the University Children's Hospital Zurich between 2017 and 2018 (ClinicalTrials.gov Identifier: NCT03059511). Briefly, infants aged 29 days to 3 months with a minimum body weight of 3.0 kg and fever without a source requiring interventional pain management for diagnostic procedures were eligible, while preterm infants with kidney or liver disease were excluded.

After parental informed consent was obtained, study participants were alternately allocated to either 0.05 mg/kg IV or 0.1 mg/kg intranasal nalbuphine (Nalbuphin OrPha® 20 mg/2 ml, OrPha Swiss, Kuesnacht, Switzerland). The relative intranasal dosage was based on intranasal bioavailability reported for other opioids; according to lipophilicity and molecular weight we expected nalbuphine intranasal bioavailability between 50 and 80% (15, 19). It should be noted that the Swiss health authority Swissmedic defined the upper limit of nalbuphine doses to be investigated in our pediatric study (0.05 mg/kg IV compared to 0.1 mg/kg intranasal nalbuphine). To create ideal conditions to enhance nasal absorption, a nasal device [Mucosal Atomization Device (MAD 300) Teleflex, USA] was used to atomize the drug particles and each nostril was cleaned before drug administration. With the expected volume of 0.03–0.1 ml we expected minimal run-off (14, 15, 19).

Blood samples [0.5 ml blood, in line with recommendations (20)] for nalbuphine serum concentration measurement were obtained 15, 30, and 120 to 180 min post-dose. Painful diagnostic interventions, including establishment of venous access for blood sampling, urinary catheterization and lumbar puncture were carried out 5 min before, 20 and 35 min after drug administration in the IV study group and 5, 20 and 35 min after drug administration in the intranasal study group, respectively. Pain score during each painful intervention was assessed by NIPS (in-house standard) (21, 22).

### Serum Drug Analysis

Nalbuphine serum levels were measured using liquid chromatography coupled to tandem mass spectrometry. The lower limit of quantification was 0.1 mcg/L, the upper limit of quantification 2,500 mcg/L. Intra- and interday assay precision was < 8.15% and < 5.3%, respectively.

### Pharmacometric Modeling and Simulation

#### PMX Based Pharmacokinetic Analyses

Data were analyzed by population pharmacokinetic (PPK) modeling, including all study subjects for whom at least one serum concentration sample was obtained. Implausible serum concentrations were defined as rising concentrations after iv administration, or concentrations >60 mcg/L (>200 mcg/L in a sensitivity analysis), corresponding to a theoretical distribution volume of <0.83 L/kg (<0.25 L/kg respectively), i.e., much lower than estimated from previously reported volume of distribution of 3.62 ± 1.77 L/kg in children 1.5–5 years (6), and were excluded from the primary analysis.

PPK model development was conducted using the software Monolix (version 2018R2, Antony, France: Lixoft SAS, 2018). IV data was modeled separately in a first step, and then fitted simultaneously with intranasal data to estimate average intranasal bioavailability. Monolix version 2020R1 was used to refit the final model and to perform model simulations in combination with Simulx in R (R Core Team, https://www.R-project.org/). Further statistics and figures were also created using R.

#### Structural Model

Both one- and two-compartment models were tested to describe the distribution kinetics of nalbuphine after IV administration. According to literature the elimination was assumed to follow first-order elimination kinetics (4, 7). Several models were tested for description of the absorption kinetics following inclusion of intranasal data, such as simple first- and zero order absorption, with/without lag-time, using one or several transit-compartments, with/without accidental enteral absorption (23).

#### Statistical Model

Inter-individual variability of model parameters was assumed to be log-normally distributed. For the residual error, both proportional and mixed error models were tested for both iv and intranasal data.

#### Covariate Analysis

Standard allometric scaling was used to account for the expected relationship between body size and clearance (CL) and/or volume of distribution (V), respectively (fixed exponents of 0.75 and 1) (24). The association of model parameters with further potential covariates (age and gender) was guided by visual inspection of scatter plots of individual random effects vs. covariates (for parameters with small eta-shrinkage), by statistical testing (likelihood ratio test of model with vs. without covariate included) and decrease in random inter-individual variability.

#### Model Evaluation

Non-nested models were compared by their Akaike information criterion and nested models by the likelihood ratio test, based on comparison of the objective function value (corresponding to −2 × log-likelihood). Further model selection criteria were the decrease in inter-individual variability and residual error, relative standard error of parameters (target: ≤ 30% to maximal 50%) and state-of-the-art goodness of fit plots (observations vs. predictions, residual diagnostics, visual predictive check).

#### Sensitivity Analyses

Two sensitivity analyses were performed by refitting the final model to the dataset including implausible serum concentrations >200 mcg/L and >60 mcg/L (see methods section), while excluding rising concentrations after IV administration.

#### PMX Based Predictions and Simulations

For each individual, predicted maximum serum concentration (C_max_), time to reach maximum serum concentration (t_max_) (derived from individual concentration-time profile predictions) and terminal half-life (t_1/2_) (derived from individual model parameters) were calculated, and summarized by median (interquartile range IQR) for each study group.

Expected overall exposure range was summarized by median simulated concentration-time profiles with 90% prediction intervals (5 and 95th percentile) for each study group. Those prediction intervals were derived from simulations for 5,000 hypothetical individuals with identical weight distribution as in the analysis dataset, and model-predicted random inter-individual variability and residual error. In addition, median concentration-time profiles with 2- or 4- fold higher dosing were simulated assuming dose proportionality. Simulated exposure range was compared to previously proposed target concentration of 12 mcg/L (i.e. efficacy threshold) under continuous iv infusion in children >1 years for treatment of postoperative pain (4), with a special focus on duration >12 mcg/L as possible indicator of duration of effect.

#### PMX Based Exposure-Pain Response Analyses

Individual concentration-time profile predictions derived from the final PPK model were further used to derive time-matched concentration predictions to investigate the association between drug exposure and severe pain (defined as NIPS >4), and expected duration of pain relief (i.e., NIPS ≤ 4). Initial descriptive analysis included a summary and boxplots of drug exposure per intervention and treatment group (median, IQR). Data from all pain assessments under nalbuphine exposure were then pooled for a model-based analysis. Fixed and mixed-effect (random intercept) logistic regression models were applied, considering linear, log-linear and non-linear relationships (Emax- and sigmoid Emax concentration-response models) with serum nalbuphine. The predicted concentration-pain response (pharmacodynamic) curve was contrasted for model evaluation with a non-parametric regression line (loess). The predicted pain response-over-time (pharmacokinetic/pharmacodynamic) curve was evaluated by comparison of predicted proportions with severe pain under median drug exposure (and 5/95th percentiles, according to PPK model predictions) with observed proportions of severe pain (mean, 95% confidence interval; all patients with NIPS assessments included irrespective of concentration measurements).

## Results

### Data

Out of 52 study subjects who were included in the study and received nalbuphine, 48 study subjects (92%) had at least one serum concentration measured. According to criteria specified above, concentrations from 10 study subjects were excluded from the primary PPK analyses: three concentrations because rising values after iv administration and seven implausible concentrations (>60 mcg/L; see definition in methods section). As such a total of 38 study subjects were retained for primary PPK analysis and exposure-pain response analysis. Table 1 shows patient characteristics (flowchart is provided as Supplementary Figure 1). Considering detailed patient numbers per intervention with available NIPS assessments under nalbuphine exposure, assessments of 20 patients during establishment of venous access (intranasal group only), 30 patients during urinary catheterization and 21 patients during lumbar puncture (intranasal and IV group combined) were available for exposure-pain response analysis. Corresponding NIPS assessments for calculation of observed proportions with severe pain were available for a total of 21 study subjects during establishment of venous access (intranasal group), 42 study subjects during urinary catheterization, and 25 study subjects during lumbar puncture (25).

**Table 1:** Characteristics of study subjects included in primary population pharmacokinetic and exposure-response analysis.

**Group**	**Nalbuphine 0.05 mg/kg iv**	**Nalbuphine 0.1 mg/kg intranasal**	**All, iv**	**All, intranasal**
Number of study subjects	15	23	26	26
Male	9 (60%)	14 (61%)	16 (62%)	16 (62%)
Age [days]	56 (37–68.5)	55 (39–62.5)	56 (40–70)	55 (39–63)
Weight [kg]	4.7 (4.3–6.0)	5.0 (4.7–5.5)	5.0 (4.5–5.9)	5.0 (4.7–5.5)

*Continuous variables are given as median (IQR), categorical variables as number (percent)*.

### PMX Modeling and Simulation

#### PMX Based Pharmacokinetic Analyses

IV nalbuphine data were well described by a two-compartmental model with the peripheral compartment volume fixed to 2 × central compartment volume (V1) as reported by Jaillon et al. (6), and weight-based allometric scaling (26). Given weight distribution in dataset, weight was centered to 5 kg. To further enhance model stability in the combined intranasal/IV model we fixed intercompartmental clearance (Q_5kg_) to 15.4 L/h (as estimated in the IV model), CL_5kg_ was estimated to 10.3 L/h and V1_5kg_ to 12.2 L. Between-subject variability was included on CL and V1 (estimated to 77 and 118%, respectively). Given available data, inclusion of age and sex as covariates did not reduce inter-individual variability nor improved model fit (*P* > 0.05) (correlation with individual random effects illustrated in Supplementary Figure 2). A simple first-order absorption model was utilized to describe intranasal absorption kinetics [rate constant (k_a_) estimated to 0.81/h]. Lag-time, one or several transit-compartments did not improve the model fit. Intranasal bioavailability was estimated to be 0.41 (95%CI: 0.26–0.56).

Sensitivity analyses (details: Table 2) resulted in similar estimates for bioavailability (0.47 to 0.36, with overlapping 95% CI). In sensitivity analysis I, CL/V1 estimates were 60/63% lower with largely increased inter-individual variability. In sensitivity analysis II CL/V1 estimates remained within the 95%CI of the reported estimates. Visual predictive checks for model evaluation are provided as Supplementary Figure 3.

**Table 2:** Population pharmacokinetic parameter estimates of the primary analysis, as well as sensitivity analyses.

	**Primary analysis** **(*****n*** **= 38 individuals)**		**Sensitivity analysis I (all data, except rising concentration after iv administration)** **(*****n*** **= 45)**		**Sensitivity analysis II (all data, except serum concentration > 200 mcg/L and rising concentration after iv administration)** **(*****n*** **= 42)**	
**Parameter**	**Value**	**R.s.e. [%]**	**Value**	**R.s.e. [%]**	**Value**	**R.s.e. [%]**
F	0.41 (0.26–0.56)*	18.5	0.405 (0.23–0.58)*	21.6	0.36 (0.23–0.49)*	18.2
ka [h^−1^]	0.81 (0.53–1.09)*	17.6	0.684	23	0.744	24.1
CL [L/h] for 5 kg infant	10.3 (6.2–14.4)*	20.2	4.6 (0.98–8.2)*	40.2	8.86 (5.4–12.3)*	20
V1 [L] for 5 kg infant	12.2 (5.8–18.6)*	26.7	5.02 (0.98–9.1)*	41.1	8.36 (3.7–13.0)*	28.5
V2 [L]	Fixed to 2 × V1		Fixed to 2 × V1		Fixed to 2 × V1	
Q [L/h] for 5 kg infant	15.4 fix		15.4 fix		15.4 fix	
omega_CL	0.679 (CV = 77%)	21.9	1.72 (CV = 427.4%)	14.5	0.718 (CV = 82.1%)	19.7
omega_V1	0.936 (CV = 118%)	15.3	2.12 (CV = 940.8%)	12.2	1.16 (CV = 168.5%)	14
Res. Error	0.353	10.1	0.396	10.2	0.39	10.6

*R.s.e., relative standard error; F, Bioavailability; ka, rate constant; CL, Clearance; V1, central compartment volume; V2, peripheral compartment volume; Q, intercompartmental clearance; omega, standard deviation of random-effect; CV, coefficient of variation (inter-individual variability); calculated as $\sqrt{\exp^{omega^{2}}-1}\;$Res. error, residual error (intra-individual variability)*;

* *95% Confidence Interval*.

#### PMX Based Predictions and Simulations

Median (IQR) individual C_max_ was 3.4 (3.0–4.8) mcg/L after intranasal administration [at t_max_ = 50 (39–64) min] vs. 17.9 (7.5–32.8) mcg/L after intranasal administration (at time = 0 h). Median terminal t_1/2_ was estimated to be 3.3 h (IQR: 2.1–6.1 h), the initial t_1/2_ to be 0.12 h (0.09–0.29 h).

**Figure 1:**
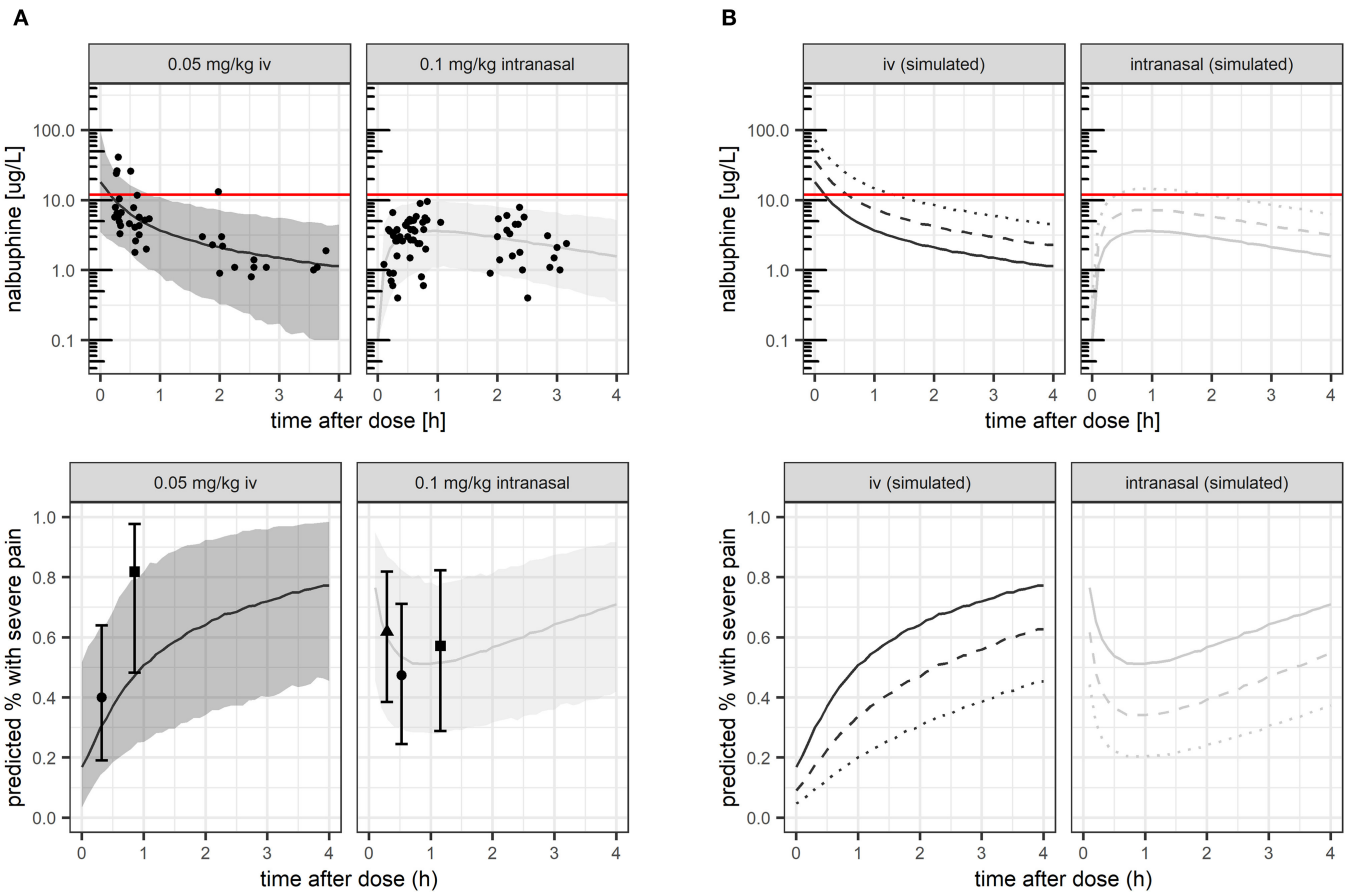
Nalbuphine exposure (**A,B** upper panels) and corresponding proportion of patients with severe pain (**A,B** lower panels). **(A)** studied nalbuphine dose of 0.05 mg/kg IV and 0.1 mg/kg intranasally, respectively. **(B)** studied nalbuphine dose compared with simulated 2–4 × higher dose. *Lines*: model-predicted median (s*olid*: studied dose, *dashed*: 2 × higher dose, d*otted*: 4 × higher dose). *Dots*: observed exposure (upper panel **A**) and observed proportion with severe pain, respectively (lower panel A, shown with 95% confidence intervals). *Shaded area*: 5 and 95th exposure percentiles.

Figure 1A (upper panel) shows model-predicted median concentration-time profiles and 95% prediction intervals, which matched the observed concentrations well. The expected median concentration following 2- or 4-fold higher dosing is shown in Figure 1B (upper panel). The proposed target concentration of 12 mcg/L was exceeded after IV administration by the median predicted exposure for 6 min after the studied dose of 0.05 mg/kg (solid line), for 30 min after double dose of 0.1 mg/kg (dashed line), and for 80 min after 4-fold dose of 0.2 mg/kg (dotted line). Following intranasal administration, the proposed target was exceeded by the median predicted exposure only after 4-fold increased dose (dotted line), between 30 and 100 min.

#### PMX Based Exposure-Pain Response Analyses

Table 3 gives a summary of drug exposure during the different medical interventions in study subjects with low-moderate (NIPS ≤ 4) vs. severe pain (NIPS > 4). Exposure tended to be higher during establishment of IV access (*P* = 0.052) and urinary catheterization (*P* = 0.189) in study subjects with low-moderate pain than in study subjects with severe pain, but less during lumbar puncture (*P* = 0.885).

**Table 3:** Summary of model-predicted individual nalbuphine concentration during the different medical interventions (establishment of venous access, urinary catheterization and lumbar puncture) in study subjects with low-moderate (NIPS ≤ 4) vs. severe pain (NIPS > 4).

	**Median [IQR] serum concentration nalbuphine (mcg/L) Patients with NIPS ≤ 4**	**Median (IQR) serum concentration nalbuphine (mcg/L)** **Patients with NIPS >4**	***p*** -value*
Establishing venous access (intransal group only)	2.93 [2.53, 3.63] *n* = 7	1.81 [0.73, 2.78] *n* = 13	0.052
Catheterization	3.23 [2.76, 9.11] *n* = 19	3.24 [1.65, 6.10] *n* = 11	0.189
Lumbar puncture	4.17 [3.16, 5.02] *n* = 8	4.12 [2.92, 5.43] *n* = 13	0.885

*n, number of study subjects*.

* *Wilcoxon-test*.

**Figure 2:**
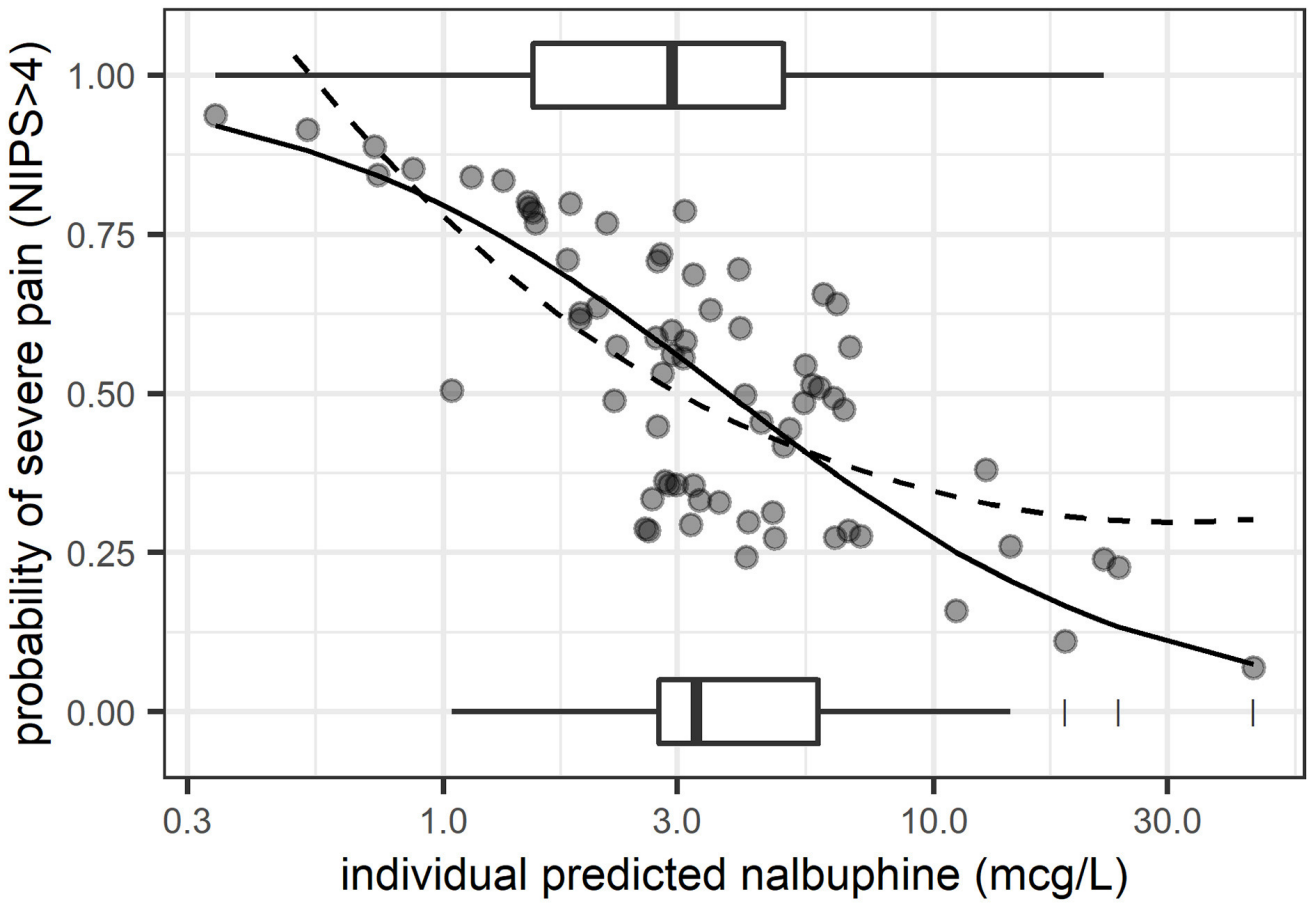
Predicted probability of severe pain (NIPS > 4) from logistic regression (random intercept model) vs. plasma exposure. *Solid line*: mean model prediction. *Dashed line*: observed mean proportions as captured by a non-parametric regression line (loess). *Boxplots*: exposure distribution for observations with severe pain (plotted at a probability of 1) and those with low to moderate pain (plotted at a probability of 0). *Dots*: individual predicted probabilities.

A logistic random intercept model with a log-linear concentration-pain response relationship on the logit scale described the pooled data best (lowest bias over concentration and over time, *P* < 0.05). The corresponding predicted probability of severe pain (NIPS > 4) vs. plasma exposure is contrasted with observed mean proportions (as captured by a loess) in Figure 2. The baseline probability of severe pain (NIPS > 4) at a concentration of 1 mcg/L was predicted to 79.5% (95% CI: 54–96%; interindividual variability expressed as ±standard deviation of estimated random effects = 53.8–92.8%), the odds ratio for doubling nalbuphine exposure was estimated to 0.49 (95% CI: 0.21–0.86). Non-linear exposure-response relationships could not be estimated with good confidence.

Pharmacometric simulations illustrating predicted probability of severe pain over time, compared with observed proportions under the given dose for model evaluation are shown in Figure 1A (lower panel). Model-based simulations for 2- or 4-fold higher doses are shown for predicted median exposure (Figure 1B). At median predicted C_max_ values, the mean probability of severe pain would be predicted to 17% (IV, at *t* = 0 h) and 53% (intranasal) under the studied dose, respectively, decreasing by 2-fold/4-fold higher doses to 9%/5% (IV), and 36%/22% (intranasal).

## Discussion

This is the first study reporting population pharmacokinetics of nalbuphine in infants 1–3 months of age, characterizing IV and intranasal kinetic profiles after single doses of 0.05 and 0.1 mg/kg, respectively. The model allowed to derive individual kinetic predictions for investigation of exposure-pain response associations, and to evaluate the potential benefit of 2-or 4-fold higher dosing by pharmacometric simulations. Output from this simulation suggested that doubling studied intranasal dose with respect to IV dosing may not be sufficient to achieve optimal pain control, despite a relative bioavailability estimated to be close to 50% (point estimate: 41%, 95%CI: 26–56%). Due to different kinetic profile after intranasal administration, simulations indicate that intranasal doses of 0.4 mg/kg would be required to exceed a previously proposed target concentration in 50% of the patients, achieved *after* 30 min of drug administration. On the other hand, a standard IV dose of 0.1 mg/kg is expected to exceed this target concentration *within* the first 30 min after drug administration. This finding has important implications for optimal timing of interventions and supports clinical usefulness of current IV doses of 0.1–0.2 mg/kg in this age group. Our PPK model parameters are consistent with previous pediatric and adult papers (4, 6, 27). For example, our average weight-normalized CL estimate would calculate to 2.28 L/kg/h (for a 5 kg infant) which is close to estimates of 2.89 L/kg/h [reported for 1.5–5 year old children (6)] to approximately 3.0 L/kg/h [1-year old infants, (4)], but significantly higher than reported in neonates (0.5 L/kg/h) (5). This suggests that maturation of nalbuphine metabolic pathways is almost complete at the age of 1–3 months. Human *in vivo* experiments had shown that the ratio of metabolite production in nalbuphine *via* CYPs and UGTs is ~23:77 (11). However, no general developmental pattern for the individual UGT isoforms is currently available. UGT2B7 activity seemed to reach adult values between 2 and 6 months, which is more or less in accordance with our assumption (28). In addition, no age-effect could be observed in our analysis, with the possible limitation of small patient numbers. That our weight-normalized clearance is slightly higher than 1.78 L/kg/h reported in young men (6) is expected for a drug with clearance well characterized by standard allometric scaling, an approach that has successfully been used for nalbuphine previously (4). In fact, our scaled “adult” drug clearance estimate would calculate to 75 L/h for 70 kg [=10.3·(70/5)^0.75^], which is in agreement with 90 L/h (range: 49–137 L/h) reported in adults after 20 mg IV (7). Estimated intranasal bioavailability of 41% appears much higher than oral bioavailability reported in adults [12% Aitkenhead et al. (7) or 16.4–17.4% Lo et al. (29)] (7, 29), and suggests that much of the drug after intranasal administration was well absorbed by the nasal mucosa in infants, allowing avoidance of gastrointestinal and hepatic first-pass metabolism.

Our PPK model-predicted median (IQR) individual C_max_ of 3.4 (3.0–4.8) mcg/L after intranasal administration at t_max_ = 50 (39–64) min was similar to measured C_max_ of 4.5 (3.5–5.6) mcg/L after 37 (32–65) min previously reported (25). The estimated bioavailability of 41% further confirms the previous observation that 2-fold higher intranasal than IV doses result in similar exposure coverage in terms of area under the concentration time curve (AUC). Our model-based concentration prediction after IV administration however shows large differences between median measured “C_max_” after 15 min (6.5 mcg/L) and actually expected median C_max_ immediately after IV dosing (17.9 mcg/L). It also revealed that even a 4-fold higher intranasal dose (i.e., 0.4 mg/kg intranasal) is not expected to achieve observed C_max_ comparable with an IV dose of 0.05 mg/kg.

An IV dose of 0.1 mg/kg is exceeding a previously proposed target concentration of 12 mcg/L (efficacy threshold) for at least 30 min. To achieve such target exposure an intranasal dose of 0.4 mg/kg is required. Performed simulations allowed us to identify an optimal time frame for interventions. Target concentrations are achieved during the first 30 min after 0.1 mg/kg IV administration, whereas efficacy threshold is exceeded 30 to almost 120 min after 0.4 mg/kg intranasal administration. The duration of analgesic effect was not formally assessed in our study, but pharmacokinetic and pharmacodynamic indicators of duration of effect [duration of concentration > 12 mcg/L (4) and duration of NIPS ≤ 4, respectively] could be derived by model-based simulations. In line with this model-based simulations and target exposure, exposure response associations suggested greater benefit of intranasal dose increase compared to IV dose increase. Potential maximal reduction of probability of severe pain was up to −31% following 0.4 mg/kg intranasal dosing vs. up to −12% with 0.2 mg/kg IV dosing.

Our PMX study has a several limitations. With this model-based analysis, we cannot conclude about the safety of simulated higher intranasal doses up to 0.4 mg/kg; the safety and tolerability of studied doses was not subject of this analysis, but is reported separately, manuscript submitted (25). In our PPK model not all distribution parameters could be estimated from data given tailored sampling design in young children. Incorporating literature values resulted however in unbiased model predictions with suitable extrapolation properties as discussed above. Inter-individual pharmacokinetic variability was large (CL = 77% and V1 = 118%) despite including weight as covariate for allometric scaling, and could not be further explained by age and/or sex. While this may point to almost mature metabolic pathways as discussed above, it must be noted that included infants were in a similar physiologic age group and of course prepubertal, and variability associated with immature metabolic pathways can hence not fully be excluded. As discussed separately (25), large variability and unusually high drug concentrations may partly be attributed to practical considerations, like imprecision of dosing associated with small drug volumes (between 0.02 and 0.10 ml), possible intranasal swallowing with intranasal drug administration and likely variable line flushing in context of IV drug administration. This illustrates some of the practical challenges of intravenous and intranasal drug administration in infants. Also pharmacodynamic inter-individual variability was large. This is pharmacologically not uncommon, but also here practical considerations need to be considered, as pain assessments in nonverbal children, especially infants are challenging, and may be subject to inter-observer variability (22). NIPS measured can only be considered as a surrogate indicator of pain, as other distress like hunger or positioning may falsely be interpreted as pain by the score. Use of alternative scores such as the Neonatal Facial Coding System (NFCS) may have been of interest (22), but would have decreased feasibility of the study as not standardly used in our emergency department. Also pooling NIPS assessments under different interventions may further have contributed to intra-individual variability. In this context we acknowledge the exploratory nature of the pharmacodynamic analysis, as the study was not specifically designed to investigate exposure-response relationships. As such we did not evaluate potential benefits of more complex modeling strategies, e.g., to account for a possible “placebo effect” in the absence of nalbuphine exposure in the IV group during establishment of venous access. Still, despite these limitations and large variability, a pharmacologically plausible trend toward higher exposure in patients with mild to moderate NIPS was observed during different interventions, which was significant after pooling data for exposure response analysis. We may note again that we had initially planned to study a usual dose of 0.1 mg/kg iv (as compared to 0.2 mg/kg intranasal), which was however refused by the Swiss medical authority due to safety concerns. The relatively low dose studied (0.05 mg/kg IV) may have finally facilitated the detection of a significant exposure-response relationship, suggesting optimal clinical efficacy and plateau effect at usual iv doses of 0.1–0.2 mg/kg.

This is the first study characterizing intranasal and IV population pharmacokinetics in infants 1–3 months, including evaluation of target exposure achievement and exposure-pain response associations. While relative bioavailability of intranasal nalbuphine is close to 50%, a different kinetic profile requires a higher intranasal dose of 0.4 mg/kg to achieve target exposures observed with intravenous doses of 0.1–0.2 mg/kg. A clinically relevant finding is that painful interventions are best done within first 30 min after IV administration, while with intranasal administration such interventions should be scheduled at least 30 min after dosing. Because the proposed 4 fold intranasal dose of 0.4 mg/kg may also increase the risk of adverse drug reactions, additional clinical studies are warranted to confirm these recommendations in this vulnerable pediatric patient population.

## Data Availability Statement

The datasets generated during the current study are available from the corresponding author on reasonable request.

## Ethics Statement

The studies involving human participants were reviewed and approved by Local Ethics Committee Swissmedic. Written informed consent to participate in this study was provided by the participants' legal guardian/next of kin.

## Author Contributions

MPfif: conceptualization/design, methodology, investigation, data curation, investigation, formal analysis, and writing—drafting the initial manuscript. EB-O: conceptualization/design, methodology, data curation, investigation, supervision/oversight, resources, and writing—review or editing of the manuscript. PV: conceptualization/design, funding acquisition, methodology, supervision/oversight, resources, and writing—review or editing of the manuscript. MPfis: conceptualization/design, funding acquisition, methodology, supervision/oversight, resources, and writing—review or editing of the manuscript. VG: supervision/oversight, data curation, investigation, formal analysis, and writing—review or editing of the manuscript. All authors contributed to the article and approved the submitted version.

## Funding

This study was supported by internal funds of the University Children's Hospital Zurich.

## Conflict of Interest

The authors declare that the research was conducted in the absence of any commercial or financial relationships that could be construed as a potential conflict of interest.

## Publisher's Note

All claims expressed in this article are solely those of the authors and do not necessarily represent those of their affiliated organizations, or those of the publisher, the editors and the reviewers. Any product that may be evaluated in this article, or claim that may be made by its manufacturer, is not guaranteed or endorsed by the publisher.

## Abbreviations

IV: intravenous
PMX: pharmacometric
PPK: population pharmacokinetics
NIPS: neonatal infant pain score
CL: clearance
Q: intercompartmental clearance
V: volume of distribution
V1: central compartment volume
C_max_: maximum serum concentration
t_max_: time to reach maximum serum concentration
t_1/2_: terminal half-life
AUC: area under the concentration time curve
IQR: interquartile range
k_a_: rate constant
CYPs: cytochrome P450s
UGTs: UDP-glucuronosyltransferases.
