# Supplementary material for: Pharmacometric Analysis of Intranasal and Intravenous Nalbuphine to Optimize Pain Management in Infants
Source: Front Pediatr. 2022 Mar 2;10:837492. doi: 10.3389/fped.2022.837492 (PMC8926166; doi:10.3389/fped.2022.837492)
Supplement: Supplementary file 1 [file Data_Sheet_1.docx]

Supplementary Material


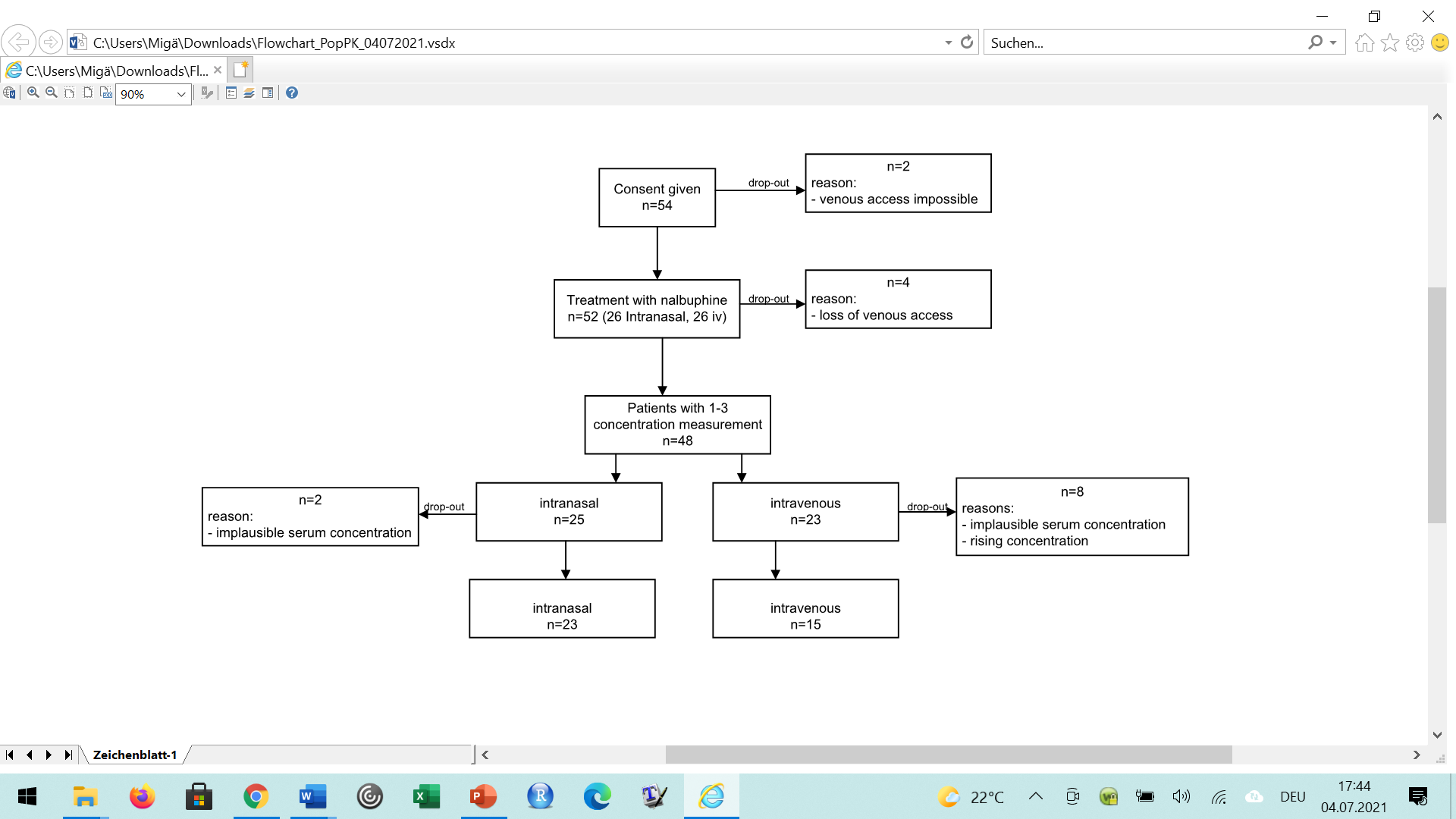


Supplementary Figure 1: Flowchart


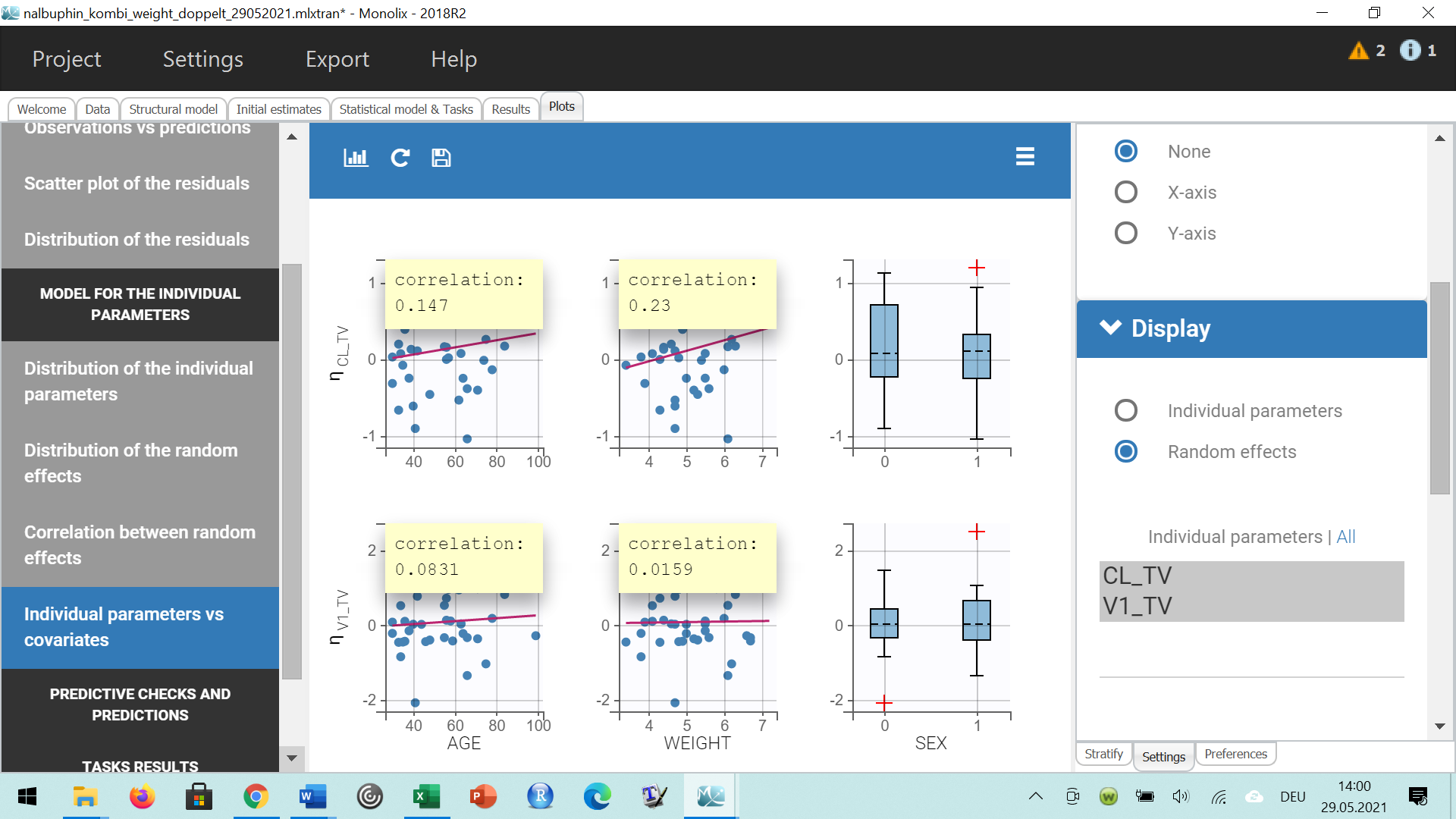


**Supplementary Figure 2:** Individual random effects ŋ for CL and V1 for nalbuphine over age, weight and sex. None of the reported correlations showed a statistically significant trend or bias (P>0.05).

| **A**  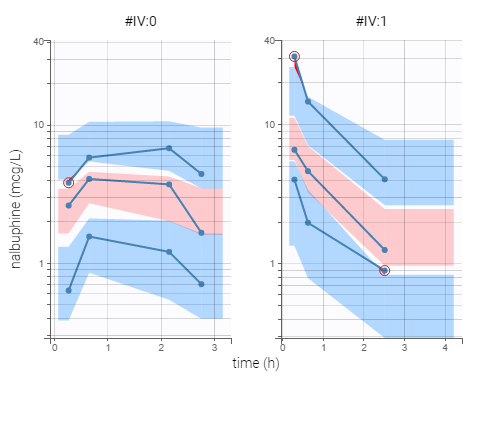 | |
| --- | --- |
| **B**  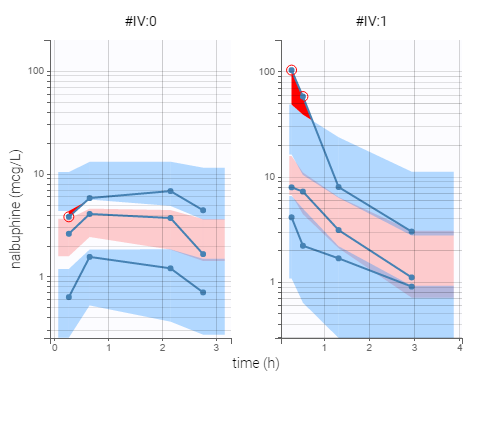 | **C**  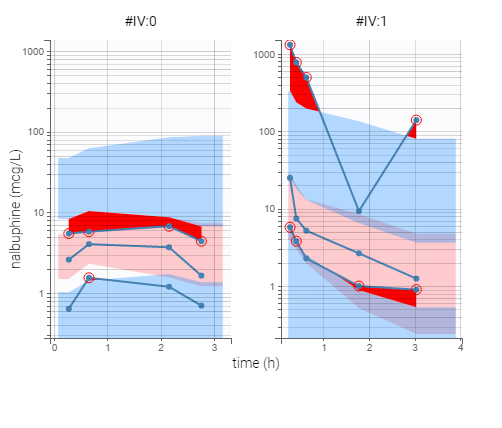 |

Supplementary Figure 3: Visual predictive checks for the mentioned models. On the y axis the logarithm of the concentration of nalbuphine in mcg/L is shown and on the x axis the time after drug administration in hours. On the left side the results for intranasal data are illustrated (#IV:0) and on the right side the results for iv data (#IV:1). The lines represent empirical percentiles (10^th^, 50^th^ and 90^th^ percentiles) and shaded areas indicate the 95%CI of respective simulated percentiles. Red areas indicate that empirical percentiles are outside the simulated percentiles. A: Primary analysis. B: Sensitivity analysis II (all data, except serum concentration > 200 mcg/L and rising concentration after iv administration) C: Sensitivity analysis I (all data, except rising concentration after iv administration).

**Code Final Model**DESCRIPTION: Nalbuphine combined IV (ADMINISTRATION ID=1) and intranasal (ADMINISTRATION ID=2) population pharmacokinetic model (2-comparmental distribution model)
[LONGITUDINAL]
input = {F1, ka, CL_TV, V1_TV, Q_TV, WEIGHT}
WEIGHT = {use = regressor}
EQUATION:
CLwt = CL_TV*(WEIGHT/5)^0.75
V1wt = V1_TV*(WEIGHT/5)^1
Qwt = Q_TV*(WEIGHT/5)^0.75
V2wt = 2*V1_TV
k = CLwt/V1wt
k12 = Qwt/V1wt
k21 = Qwt/V2wt

PK:

compartment(cmt=1, amount=Ac)
peripheral(k12,k21)
oral(type=2, cmt=1, ka=ka, p=F1)
iv(type=1, cmt=1)
elimination(cmt=1, k=k)
Cc = Ac/V1wt
OUTPUT:
output = Cc
